# Prevalence of chronic viral hepatitis infections in Karaj, Iran

**DOI:** 10.11604/pamj.2017.28.186.10269

**Published:** 2017-10-30

**Authors:** Kourosh Kabir, Hassan Hoseini, Mohammad Miri, Fatemeh Amrollahi, Elham Bahraini, Parviz Afrogh, Enayatollah Kalantar

**Affiliations:** 1 Social Determinants of Health Research Center, Alborz University of Medical Sciences, Karaj, Iran; 2 Blood Transfusion Researcher Center, High Institute for Education and Research in Transfusion Medicine, Tehran, Iran; 3 Karaj Regional Blood Transfusion Center, Karaj, Iran; 4 Biochemistry Department of Medicine Faculty, Iran University of Medical Sciences, Tehran, Iran; 5 Mycobacteriology and Pulmonary Research Department, Pasteur Institute of Iran, Tehran, Iran; 6 Department of Microbiology and Immunology, School of Medicine, Alborz University of Medical Sciences, Karaj, Iran

**Keywords:** Hepatitis B, Hepatitis C, blood safety, Iran, prevalence

## Abstract

**Introduction:**

Viral hepatitis is challenging for health and blood safety. Studies carried out on blood donors can help find the frequency and trending of hepatitis B and C infections in a community and also safety of donation. The study aim is to determine the prevalence of HBV and HCV in Karaj blood donors over a four year period between 2010 to 2013.

**Methods:**

This study reports the results of a cross sectional seroepidemiological study of hepatitis B and C in blood donors. Data on hepatitis infection and demographic characteristics of donors were gathered from blood donor registries. Frequency of hepatitis infections were described with 95% confidence interval. Chi square and logistic regression were used for analysis.

**Results:**

The frequency of HBV and HCV infection in Karaj blood donors was 0.40% and 0.18% respectively. In first time donors, HBV and HCV positivity risk was respectively 3.59 and 4.8 fold in people with primary education (OR=3.59; 95% CI between 2.68-4.80) comparing to academic level. Frequency of hepatitis B has decreased significantly (P<0.001) during study period but frequency of Hepatitis C has not changed significantly.

**Conclusion:**

The frequencies of HBV and HCV infection in Karaj blood donor population is low. There are equal infection rates within both genders. This must be considered in controlling transmission of infection in this area.

## Introduction

Infections due to hepatitis is are a major public health concern all over the world [1, 2]. Individuals with chronic infections have a higher risk of developing liver cirrhosis and hepatocellular carcinoma. This has an important role in disease burden [3-5] and there is a significant financial cost for managing the problem [6]. Blood donation has an important role in the transmission of viral hepatitis particularly hepatitis B and C and is considered a significant challenge to blood safety [7]. The world health organization estimates that over 2 billion people have been exposed to the hepatitis B virus. Approximately 350 million people are chronically infected with HBV [8] and 780 000 people die every year due to complications of hepatitis B [3]. Infected people or asymptomatic carriers are the only reservoir of hepatitis B and C infection. Prevalence of HBsAg carriers in the world has been reported from 0.1% - 20% with high percentage in tropical countries [9-13]. Theoretically the elimination of hepatitis B and c is possible. There are new and accurate strategies for screening of donated blood [7, 14] preventing transfer to others. There are safe vaccines for hepatitis B and effective treatments for chronic infections. The underlying problem lies with the differing approach and strategies by countries based on their resources and access to them, hence the problem continues. There are different studies on the frequency of HBV and HCV in blood donors all over the world which show different prevalence's [9-13, 15]. These studies have shown the frequency and trend of hepatitis B and C infections in the different societies and also a way to show the safety of donation. The Frequency of infected people with hepatitis B and C differs based on region, study type and when the study was carried out [13, 15-18]. Karaj is the capital city of the Alborz Province which is located west of Tehran and has more than 2 million inhabitants in the 2011 census being the fourth most populous city of Iran. There is little information on hepatitis infection in Karaj. Each year about 50,000 residents donate blood giving the opportunity to study the blood borne diseases in the region. The study aims to determine the prevalence of HBV and HCV in Karaj blood donors over a four year period from 2010 to 2013.

## Methods

This study reports the results of a cross sectional seroepidemiological study of hepatitis B and C in blood donors of Karaj within a 4 year period from March 2010 - 2013. The blood transfusion organization registries were used for data collection. During the study period 202814 people donated in Karaj. 67828 people were first time donors whilst 134986 people were frequent donors. Multiple donation information was removed from the study by using frequent donors last donation for the analysis. Based on the screening protocol for blood donation in Iran, individuals who were at least 16 years old and in a healthy state were eligible for blood donation. Blood donors were also screened for high risk behaviors by a questionnaire and people with high risk behaviors such as drug use and having multiple partners were not eligible for blood donation. The selected study group went through a blood donation process and a sample of their blood was sent for screening to identify blood infections like hepatitis B and C. All the samples were tested by standard operating procedure (SOP) of Iranian blood transfusion organization. Third and fourth generation ELISA kits respectively were used for screening of HBV and HCV and Confirmatory tests were done for reactive samples of donations. Data on blood donors transferred to SPSS V.19 software. Frequency of hepatitis B and C were described with numbers and percentages within 95% confidence interval. Chi square was used to compare categorical variables. Multivariate logistic regression (using backward Stepwise-Wald method) was used to evaluate demographic variables with Hepatitis B and C in first time donors. Results of logistic regression are presented as OR and 95% CI. Formal written consent was given by blood donors as a part of donation protocol. This study had been approved by the ethics committee of Alborz University of Medical Sciences.

## Results

During the study period (March 2010 to 2013) a total of 202814 donations were carried out by 109817 donors. All donations were screened; among which 193 people (0.18%) were positive for HCV and 444 people (0.40%) for HBV. Most of the study group were male (93.7%); moreover 78.6% were married and 27.2% of them had university degrees. Some of the study samples had repeated donations (38.2%) while 61.8% were first time donors (Table 1). Table 1 also shows the frequency of HBsAg positive in relation to gender, age groups, educational level, marital status and history of blood donation. There was no significant difference between male and female HBsAg positive rates but were higher in older age groups (P<0.001). Donors with lower educational levels had higher frequencies of Hepatitis B infection (P<0.001). Married people (P<0.001) and first time donors (P<0.001) also showed higher Hepatitis B infection rates.

**Table 1:** Frequency of Hepatitis B and C infection in Karaj blood donors and in relation to some of demographic factors

Variable	Donors Population		Hepatitis B Positive	P Value	Hepatitis C Positive	P Value
	Number (%)		Number (%)		Number (%)	
**Gender**						
Male	102895	93.70%	417(0.40%)	0.846	186(0.180%)	0.126
Female	6924	6.30%	27(0.39%)		7(0.10%)	
**Age(Years)**						
15-24	13060	11.9%	46(0.35%)	<0.001*	8(0.06%)	0.011*
25-34	43150	39.3%	135(0.31%)		79(0.18%)	
35-44	30736	28%	129(0.42%)		66(0.21%)	
45-54	17706	16.1%	100(0.56%)		33(0.19%)	
≥55	5166	4.7%	34(0.66%)		7(0.13%)	
**Education**						
≤Primary	11737	10.7%	120(1/02%)	<0.001*	38(0.32%)	<0.001*
High school	22142	20.2%	126(0.57%)		64(0.28%)	
Diploma	45994	41.9%	118(0.26%)		71(0.15%)	
University Degree	29892	27.2%	79(0.26%)		21(0.7%)	
**Marital Status**						
Married	86096	78.6%	390(0.45%)	<0.001*	154(0.18%)	0.680
Single	23475	21.4%	54(0.23%)		39(0.17%)	
**Blood Donor**						
First time	67828	61.8%	431(0.63%)	<0.001*	185(0.27%)	<0.001*
Frequent	41991	38.2%	13(0.03%)		8(0.02%)	
**Total**	109819	100%	444(0.40%)	---------	193(0.18%)	------

* p value < 0.05 considered as significant

Note: In some parts of table, sum of numbers are not compatible with total numbers because of missing data.

**Figure 1:**
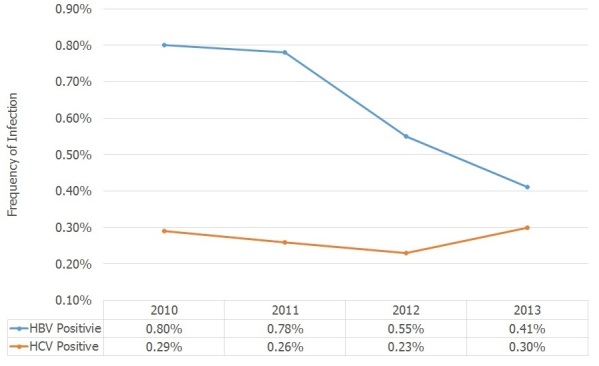
Trend of hepatitis B and C infection in Karaj first time blood donors from 2010 to 2013

Table 2 shows the relationship between HBsAg with demographic variables in first time donors. Age, sex, education and marital status was entered into the model. After adjustment, education and marital status were related to being HBs Ag positive. The risk of being HBs positive in people with primary education and high school education was 3.59 (OR=3.59; 95% CI: 2.68-4.80) and 2.05 (OR=2.05; 95% CI: 1.55-2.73) times greater respectively than people with a university degree Single people were at lower risk to have Hepatitis B infection (OR=0.69; 95% CI: 0.51-0.93). Table 3 shows the relationship between Hepatitis C infections (HCV) with demographic variables in first time donors. Age, sex, education and marital state was entered into the model. After adjustment; education and gender were associated with Hepatitis C infection. Comparing people with university degrees the risk of being HCV positive was 4.8 times greater in people with primary education (OR=4.81; 95% CI: 2.82-8.21) and 4.1 times greater in people with high school education level (OR=4.07; 95% CI: 2.48-6.68) and 2.21 times in people with diploma (OR=2.21; 95% CI: 1.36-3.60). Male donors had higher risk of Hepatitis C infections Trend of hepatitis infection in first time blood donors of Karaj are shown in Figure 1. Frequency of hepatitis B infection has decreased significantly (P<0.001) but frequency of Hepatitis C infection has remained unchanged over the years.

**Table 2:** Adjusted Odds Ratio for Risk factors of Hepatitis B infection in blood donors

	B	S.E.	Wald	df	Sig.	Exp(B)	95% C.I. for EXP(B)	
							Lower	Upper
**Education(Degree)**			124.122	3	0.000			
**≤Primary**	1.279	0.148	74.561	1	0.000	3.594	2.688	4.805
**High school**	0.720	0.145	24.714	1	0.000	2.055	1.547	2.730
**Diploma**	-0.038	0.146	0.067	1	0.796	0.963	0.724	1.281
**(Marriage Vs single)**	-0.370	0.150	6.118	1	0.013	0.691	0.515	0.926
**Constant**	-5.470	0.214	655.078	1	0.000	0.004		

*: p value < 0.05 considered as significant

**Table 3:** Adjusted Odds Ratio for Risk factors of Hepatitis C infection in blood donors

	B	S.E.	Wald	df	Sig.	Exp(B)	95% C.I. for EXP(B)	
							Lower	Upper
**Education(Degree)**			45.850	3	0.000			
**≤Primary**	1.571	0.273	33.205	1	0.000	4.811	2.819	8.208
**High school**	1.405	0.252	31.035	1	0.000	4.074	2.485	6.678
**Diploma**	0.794	0.249	10.209	1	0.001	2.212	1.359	3.601
**Sex**	0.690	0.387	3.189	1	0.074	1.994	0.935	4.254
**Constant**	-8.614	0.794	117.716	1	0.000	0.000		

*: p value < 0.05 considered as significant

## Discussion

Out of 202814 donation during 2010-2013 (from 109817 blood donors), 444 people (0.40%) and 193 people (0.18%) were HBV and HCV positive respectively. The present study shows a low level of endemicity of hepatitis infection in Karaj blood donors´ population. The Frequency of hepatitis B and C infections over the years [2,10, 19] differs within the geographical location within the countries and varies worldwide [17, 20-23]. The Prevalence of hepatitis B and C in blood donors is a reflection of the infections in different communities and they show the same variations [8]. The frequency of hepatitis B and C in Karaj blood donors is relatively low, Comparing to the other regions of Iran, [17, 21, 24] and similarly is lower than some of other Middle Eastern countries such as Jordan which is 1.53%[14]. Our study is also compatible with low frequencies of viral hepatitis in Iranian blood donors. Kafi-abad et al., have reported a prevalence of 0.4% for HBV infection (from 1998-2007 A.C) [15] and a prevalence of 0.56% and 0.13% for hepatitis B and C (from 2004-2007 A.C) in Iranian blood donors [25]. Another study among blood donors from 2005-2011 has reported 0.39% and 0.11% frequencies for Hepatitis B and C infection respectively [13] and a study on Shiraz blood donors showed similar frequencies [26].

On the other hand, there are some population based studies which have reported higher prevalence of hepatitis B and C in Iran but these results are from studies carried out over a decade ago. A study on HBV infection of the general public in 1997 reported a frequency over 5% [20] and Abdolahi et al. in 2006 reported a 9.7% prevalence in Golestan province [17]. A systematic review by Mohammadi et al., shows a decreasing trend of hepatitis B infections especially after 2006. Due to the heterogenic nature of information used the writer suggests carrying out more accurate studies to be able to estimate the point prevalence [27] of the hepatitis B infection. Looking at the results of these studies, [13, 15, 25, 26] there is a decreasing trend of hepatitis B [19, 28] which is compatible with our study results. National vaccination program for hepatitis B which started since 1993 has been successful in decreasing the hepatitis B infection. Furthermore, programs for controlling mother to child transmission, safer blood transfusions and health education campaigns on hepatitis has played a preventative role. Merat et al., has shown the frequency of 0.5% (by RIBA) for hepatitis C infection [16] and another study reported 2.6% (by ELISA) and 1% (by RIBA) for hepatitis C infection in Golestan province [29]. On the other hand, our study doesn't show such a decreasing pattern of hepatitis C infection during the study period in Karaj. Men and women didn't have different positivity rates but donors who were older, had lower educational levels, were married and first time donors, had a higher frequency of HBs Ag. It was the same for hepatitis C positivity rates, except there was no significant difference based on marital status. Some studies have shown that infection rates are similar in both genders [22] but in other studies men generally have higher rates [16, 17, 30]. In our study women were at higher risk of Hepatitis B infection. This was probably due to having hepatitis B positive partners or having more high risk behaviors than other communities. This must be considered as a real public health concern [31] because of the probability of vertical transmission from mothers to child especially for hepatitis B transmissions.

There are also higher rate of infection in older people as shown in other studies [22] which could be explained by being at risk for longer period´s time and infection rates being higher in previous decades. On the other hand, having implemented national hepatitis B vaccination program since 1993 in Iran could be another important factor for lower rates of infection in younger groups [32]. According to CDC estimation about 3.9 million people worldwide are infected with HCV, with highest prevalence among the age group 30-39 years [19] which is compatible to the current study carried out on blood donors in Karaj. Blood donors with lower educational level in our study had higher rates of hepatitis B and C, a finding which is being confirmed by other studies [13, 33]. First time blood donors had higher rates of hepatitis infections compared to frequent donors, which is comparable to other studies [13, 30]. This is a result of screening method used for blood safety checks in their first donation. Safety of blood products is important especially in platelets and factors [7, 14]. The low rates of hepatitis infections in Karaj from first time donors by the use of sensitive screening methods, results in low residual risk of transmission and the preparation of a safe blood product.

## Conclusion

It seems that chronic hepatitis B and C infection in Karaj is relatively low and blood products have low residual risk of transfusion of hepatitis infections. There are relatively equal infection rates in men and women must be considered in controlling the transmission of infection in our province.

### What is known about this topic

- Prevalence of chronic hepatitis in the world is different based on the region, preventative measures and treatment strategies;
- Prevalence of HBV and HCV in blood donors can show the frequency and trend of hepatitis B and C infections in the different societies allowing to show how safe blood donation is in the transmission of hepatitis;
- There is little information of hepatitis infection in Karaj which is the fourth most populous city in Iran with 50,000 blood donation.

### What this study adds

- It seems that chronic hepatitis B and C infection in Karaj is relatively low and blood products have low residual risk of transfusion of hepatitis infections. Sex, education and marital status were associated with being HBs Ag or hepatitis C positive. Some demographics could be used to stratify the high risk group for blood donation ;
- There are Relatively equal infection rates in men and women which may suggest the different transmission modes in Karaj compared to other regions.

## Competing interests

The authors declare no competing interests.
